# Overexpression of a bifunctional enzyme, CrtS, enhances astaxanthin synthesis through two pathways in *Phaffia rhodozyma*

**DOI:** 10.1186/s12934-015-0279-4

**Published:** 2015-06-18

**Authors:** Shuang Chi, Yanfeng He, Jie Ren, Qian Su, Xingchao Liu, Zhi Chen, Mingan Wang, Ying Li, Jilun Li

**Affiliations:** State Key Laboratories for Agro-biotechnology, College of Biological Sciences, China Agricultural University, Beijing, 100193 People’s Republic of China; Department of Applied Chemistry, College of Sciences, China Agricultural University, Beijing, 100193 People’s Republic of China

**Keywords:** *Phaffia rhodozyma*, Astaxanthin, HDCO, Astaxanthin synthase (CrtS), Overexpression

## Abstract

**Background:**

A moderate-temperature, astaxanthin-overproducing mutant strain (termed MK19) of *Phaffia rhodozyma* was generated in our laboratory. The intracellular astaxanthin content of MK19 was 17-fold higher than that of wild-type. The TLC profile of MK19 showed a band for an unknown carotenoid pigment between those of β-carotene and astaxanthin. In the present study, we attempted to identify the unknown pigment and to enhance astaxanthin synthesis in MK19 by overexpression of the *crtS* gene that encodes astaxanthin synthase (CrtS).

**Results:**

A *crtS*-overexpressing strain was constructed without antibiotic marker. A recombinant plasmid with lower copy numbers was shown to be stable in MK19. In the positive recombinant strain (termed CSR19), maximal astaxanthin yield was 33.5% higher than MK19, and the proportion of astaxanthin as a percentage of total carotenoids was 84%. The unknown carotenoid was identified as 3-hydroxy-3′,4′-didehydro-β,Ψ-carotene-4-one (HDCO) by HPLC, mass spectrometry, and NMR spectroscopy. CrtS was found to be a bifunctional enzyme that helped convert HDCO to astaxanthin. Enhancement of *crtS* transcriptional level increased transcription levels of related genes (*crtE*, *crtYB*, *crtI*) in the astaxanthin synthesis pathway. A scheme of carotenoid biosynthesis in *P. rhodozyma* involving alternative bicyclic and monocyclic pathways is proposed.

**Conclusions:**

CrtS overexpression leads to up-regulation of synthesis-related genes and increased astaxanthin production. The transformant CSR19 is a stable, secure strain suitable for feed additive production. The present findings help clarify the regulatory mechanisms that underlie metabolic fluxes in *P. rhodozyma* carotenoid biosynthesis pathways.

**Electronic supplementary material:**

The online version of this article (doi:10.1186/s12934-015-0279-4) contains supplementary material, which is available to authorized users.

## Background

Astaxanthin (3,3′-dihydroxy-β,β-carotene-4, 4′-dione), a red–orange carotenoid compound, has undergone considerable commercial development in recent decades [1] because of its usefulness in aquaculture as feed additive [2, 3] and in medicine/health care as an antioxidant reagent that reduces oxidative damage caused by reactive oxygen species (ROS) [4, 5].

The basidiomycetous yeast *Phaffia rhodozyma* (sexual form, *Xanthophyllomyces dendrorhous*) synthesizes astaxanthin as a primary carotenoid pigment and β-carotene (the precursor of astaxanthin) as a secondary abundant pigment. Astaxanthin comprises ~70% of total pigment molecules in *P. rhodozyma*, and the cells can be used directly as a feed additive [6].

Because of the commercial importance of astaxanthin, many studies have focused on mutation breeding of more productive *P. rhodozyma* strains [6, 7] or on optimization of the fermentation process [8, 9], resulting in increased astaxanthin-producing capacity [10].

**Figure 1:**
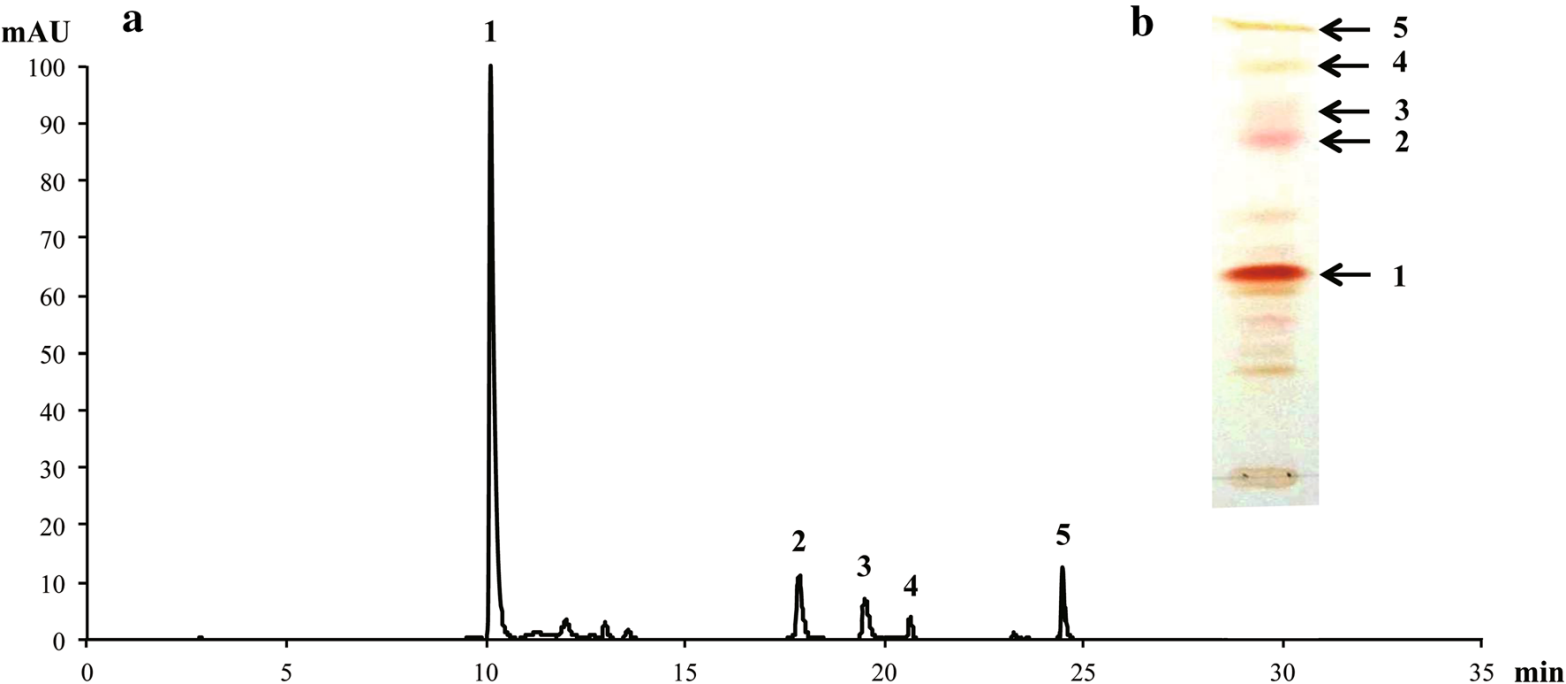
HPLC and TLC profiles of carotenoids produced by *P. rhodozyma* mutant strain MK19. Cells were cultured in flasks for 120 h. **a** HPLC profiles (detection wavelength 480 nm). **b** TLC profiles.* Peak numbers* in **a** correspond to* arrow numbers* in **b**. *1* astaxanthin; *2* unknown carotenoid; *3*,*4* keto derivatives; *5* β-carotene. Astaxanthin (66% of total carotenoids) was the predominant carotenoid. The unknown pigment (*2*), rather than β-carotene (*5*), was the second most abundant.

We previously generated a moderate-temperature, astaxanthin-overproducing mutant *P. rhodozyma* strain, termed MK19, by NTG and Co60 mutagenesis [11]. In comparison with wild-type strain JCM9042, fatty acid content was lower, optimized astaxanthin yield was ~17-fold higher, and carotenoid composition was strikingly altered in MK19. Substrate (β-carotene) and intermediate molecules (keto derivatives) were converted more efficiently to the desired end product astaxanthin in MK19; in comparison with JCM9042, the percentage of astaxanthin increased from 61 to 66%, and the combined percentage of β-carotene and keto derivatives decreased from 28 to 14%. The content of an unidentified pink/purple-colored carotenoid was 15% higher in MK19 than in JCM9042. HPLC and TLC profiles of carotenoids produced by MK19 are shown in Figure 1a, b. The peaks and arrows indicated by numbers 1–5 were identified respectively as astaxanthin, the unknown carotenoid, two keto derivatives (#3, 4), and β-carotene. The chromatographic properties and absorption spectra data of the unknown carotenoid suggested that it is an intermediate in astaxanthin production. Enhanced activity of astaxanthin synthase (CrtS) would presumably increase conversion of the unknown carotenoid and others to astaxanthin.

Astaxanthin is the most developed xanthophyll, and its antioxidant activity is higher than that of other carotenoids [1]. The goal of most *P. rhodozyma* studies is to increase production of total carotenoid pigments, particularly astaxanthin. Genetic engineering of the carotenoid biosynthesis (carotenogenic) pathway is a powerful tool for enhancing astaxanthin production [9, 12, 13].

It was unclear from previous studies whether the unknown carotenoid can re-enter the carotenogenic pathway to further increase astaxanthin production. The *crtS* gene is involved in conversion of β-carotene to xanthophylls. This multifunctional P450 monooxygenase catalyzes all steps in the pathway from β-carotene to astaxanthin by oxygenation of carbons 3 and 4 [14, 15], with the help of an auxiliary cytochrome P450 reductase (CPR) encoded by *crtR* gene which provides CrtS with the necessary electrons for substrate oxygenation [16]. In the present study, the *crtS* gene was overexpressed in an attempt to transfer accumulated unknown carotenoid and further increase astaxanthin production. Comparison of carotenoid profiles between MK19 and the target transformants allowed us to clarify the relationship between carotenoid composition and cell phenotype resulting from *crtS* overexpression.

## Results and discussion

Classical mutagenesis is typically performed as the initial strategy for overproduction of astaxanthin in *P. rhodozyma*. The mutant MK19 was selected as an astaxanthin-overproducing host strain. Astaxanthin yield can be further enhanced by genetic modification of the carotenoid synthesis pathway. We were interested by a pink/purple-colored unknown carotenoid whose level was ~15% higher in MK19 than in wild-type strain. To evaluate the potential of the unknown compound as a substrate for further production of astaxanthin through up-regulation of astaxanthin synthase (CrtS) gene, an episomal vector to achieve functional CrtS overexpression in homologous host MK19 was used and examined the effect on astaxanthin synthesis.

### Construction of CrtS-overexpressing strains

The *crtS* gene has a length of 3,167 bp, including 17 introns and 18 exons, and encodes a 62.6-kDa protein composed of 557 amino acids. We found consistent sequences in wild-type *P. rhodozyma* strain JCM9042 and mutant MK19. During cloning of CrtS cDNA, we obtained for the first time an alternative transcript in MK19 with only 114 amino acids. Lodato et al. reported that the ratio of mature mRNA to alternative mRNA for *crtI* (phytoene desaturase gene) and *crtYB* (lycopene cyclase gene) changed in response to physiological or environmental conditions [17]. The proportion of *crtS* alternative transcript may have a similar regulatory function in carotenogenesis.

To compare the function of homologous Rbs (in *P. rhodozyma crtS*) with that of heterologous Rbs (in pGBKT7 carrying that of *Saccharomyces cerevisiae*) and to achieve alternative CrtS expression levels, two lengths of *crtS* cDNA fragments were amplified and fused into the ADH1 (alcohol dehydrogenase isozyme I) promoter-terminator cassette of pGBKT7, resulting in plasmids pGBKT7-*crtSr* (containing Rbs of *crtS* gene, Additional file 1: Figure S1a) and pGBKT7-*crtS* (plasmid carrying Rbs of *Saccharomyces cerevisiae*, Additional file 1: Figure S1b). Following electroporation into MK19, ~40% of two types of cells survived. Thirty positive colonies with dark-red color were selected. Following flask culture and evaluation of cell density and total pigment content, the two best-performing transformants, termed CSR19 (containing pGBKT7-*crtSr*) and CS19 (containing pGBKT7-*crtS*), were selected for the subsequent experiments.

### Plasmid copy numbers in the transformants

Target plasmid copy numbers in CS19 and CSR19 were determined by qPCR. For separate detection of plasmid and host chromosomal DNA (*β*-*actin*), two primer sets (see Additional file 2: Table S1) specific for the plasmid pGBKT7 GAL4 DNA binding domain (DNA-BD) and *β*-*actin* (internal reference) from the *P. rhodozyma* chromosome were used. Standard curves for *β*-*actin* (Additional file 3: Figure S2a) and DNA-BD (Additional file 3: Figure S2b) were constructed by serial 10-fold dilution of the quantitative standard sample, pGBKT7-*actin*. Each standard dilution was amplified by qPCR in triplicate. For each gene, Cp values were plotted against the logarithm of known initial copy numbers (n = 2). Standard curves were generated by linear regression through these points, and had coefficient of determination (R^2^) = 0.99985 for DNA-BD and 0.99994 for *β*-*actin*.

Amplification specificity of primer sets specific for chromosomal and pGBKT7 sequences was checked by melting curve analysis (data not shown), and qPCR amplifications of total DNA of CS19 and CSR19 were performed simultaneously with standard samples. Absolute copy numbers of *β*-*actin* and DNA-BD were determined from the corresponding standard curves. Plasmid copy numbers in CS19 and CSR19 cells were calculated by dividing the copy number of DNA-BD by the copy number of *β*-*actin*. Plasmid copy numbers were 9.1 for CS19 and 8.2 for CSR19 (Additional file 3: Figure S2c). Although plasmid copy numbers were not increased, transfer >10 times of positive strains on YPD solid medium slants showed that the plasmids remained stable, indicating good compatibility between the plasmid (pGBKT7) and host cell. The lower levels of heterologous protein expressed in the host presumably did not pressure the endoplasmic reticulum and Golgi apparatus, and the plasmid could undergo stable replication in the cells. *P. rhodozyma* can be used directly as a feed additive, and we therefore do not need to consider additional markers for engineering of strains (as is the case in antibiotic resistance screening). The stability and security of the strains are promising for our applications.

### Overexpression of CrtS promotes cell growth and astaxanthin yield

CSR19, CS19, and MK19 were grown in flasks to evaluate the effect of CrtS overexpression. Aliquots were collected at 24, 48, 72, 96, and 120 h for determination of biomass production and amounts of synthesized astaxanthin. Experiments were performed in triplicate or quadruplicate.

**Figure 2:**
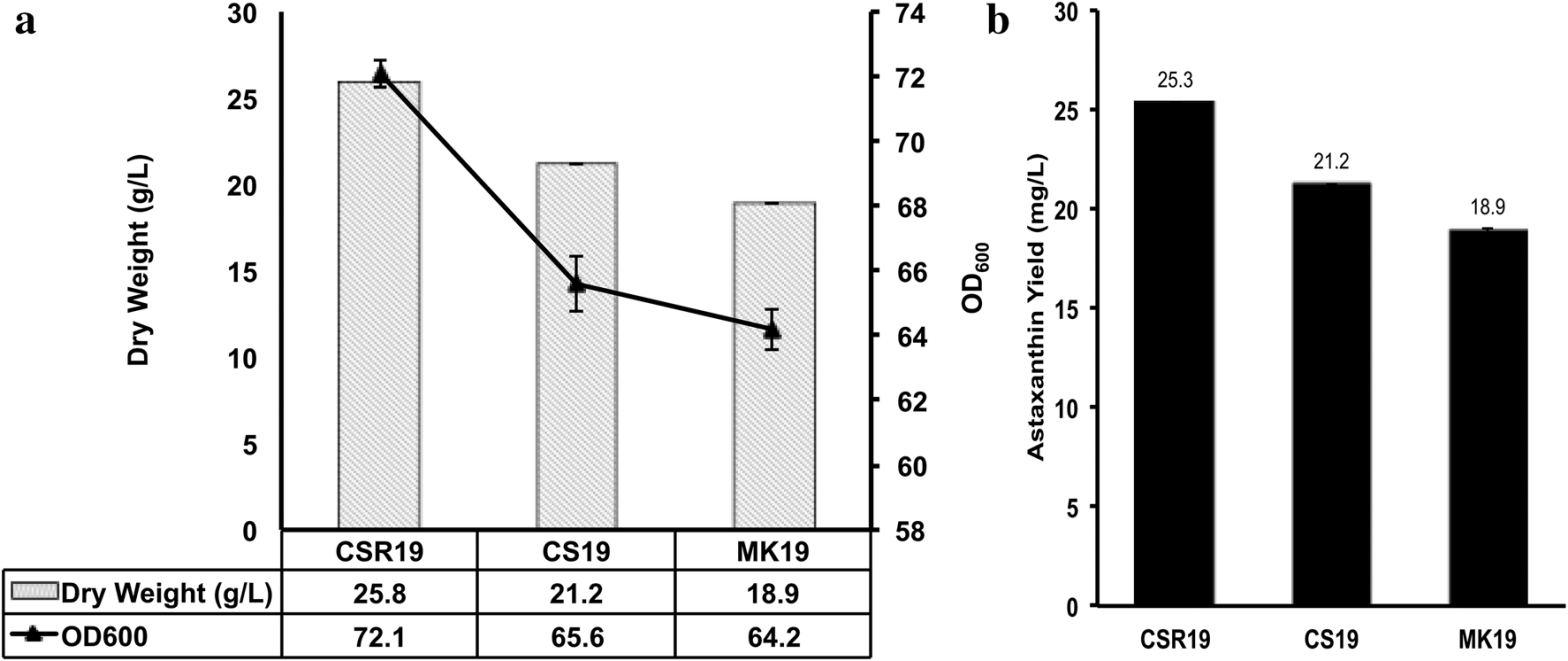
Comparative biomass and astaxanthin yields in strains MK19, CS19, and CSR19 during flask fermentation for 120 h. **a** Dry weights and OD_600_ values for the three strains. Cell dry weights were determined by centrifuging 20 mL broth at 12,000×*g*, rinsing with distilled water, and drying at 105°C to a constant weight (~10 h). Dry weights were 25.8 g/L for CSR19, 21.2 g/L for CS19, and 18.9 g/L for MK19. **b**. Astaxanthin yields (values shown are means of three individual cultures) were 25.3 mg/L for CSR19 and 21.2 mg/L for CS19 (33.5 and 11.8% higher than the value for MK19). *Error bars* SD from three independent biological replicate experiments.

Cell growth and astaxanthin yield for the three strains are summarized in Figure 2. Both these parameters were promoted in CSR19 and CS19, thereby enhancing oxidative stress tolerance. Carotenoids, being secondary metabolites, are not essential for cell survival, but do promote biomass accumulation under oxidative conditions. Cell dry weight after 120 h was enhanced by 36.5 and 12.2% for CSR19 and CS19 respectively, compared with MK19 (Figure 2a). CrtS overexpression resulted in excessive astaxanthin production. Maximal astaxanthin yield was 25.3 mg/L for CSR19 and 21.2 mg/L for CS19, that 33.5 and 11.8% higher than for MK19, respectively (Figure 2b). These results demonstrated the capacity of *P. rhodozyma* for increased carotenoid synthesis, and its potential as a “cell factory” for commercial-scale production of various carotenoids.

The effects of broth amount (6 vs. 10% filling amount) on growth and carotenogenesis of the three strains were also studied. Because of their higher carotenoid synthesis, CSR19 and CS19 were less sensitive to oxygen content in medium; biomass accumulation and carotenoid synthesis did not show difference markedly for 6 vs. 10% filling amount. Limitation of oxygen supply is often a problem in industrial fermentation; therefore, the lack of sensitivity to this parameter is an advantage for astaxanthin production.

### Adaptation of carotenoid composition to CrtS expression level

CSR19 and CS19 differ in the Rbs sequence in the upper end of expressed target gene *crtS*. The comparative study of cell growth and astaxanthin synthesis suggested that Rbs of *crtS* (harbored by pGBKT7-*crtSr*) has higher affinity with ribosomes, which results in more efficient synthesis of CrtS. Changes in metabolic flux direction were observed among carotenoid biosynthesis pathways, those indicated adaptation to differences in CrtS activity between CSR19 and CS19.

Proportions of various pigment compounds in the three strains are shown in Table 1. Relative to MK19, CSR19 had a 7% higher proportion of astaxanthin, 6% lower proportion of β-carotene, and similar proportion of the unknown carotenoid. In contrast, CS19 had similar astaxanthin, 5% lower β-carotene, and 4% higher proportion of the unknown carotenoid. These findings indicated that redundant substrates for xanthophyll synthesis (e.g., β-carotene and keto derivatives) may be further transformed to both astaxanthin and the unknown carotenoid through increased CrtS (astaxanthin synthase) activity.

**Table 1:** Composition of major carotenoids in three strains

Strain	Astaxanthin	HDCO	Keto1	Keto2	β-carotene	Other carotenoids
CSR19	73.3 ± 1.0	7.5 ± 0.6	0.6 ± 0.1	0.3 ± 0.1	0.7 ± 0.1	17.6 ± 1.8
CS19	67.2 ± 0.8	11.8 ± 0.7	1.8 ± 0.1	0.5 ± 0.1	1.8 ± 0.0	17.0 ± 0.1
MK19	66.3 ± 0.3	7.6 ± 0.3	5.3 ± 0.1	2.0 ± 0.0	6.8 ± 0.3	12.0 ± 0.3

Values shown are proportions of the given compound as a percentage of total pigments in the given strain (mean from 3 individual cultures).

Abundant CrtS may lead to maximal proportion of astaxanthin (the desired end product), whereas insufficient CrtS may lead to accumulation of the unknown carotenoid. Relative production of the various compounds regulated by CrtS clearly depend on CrtS expression level.

### Overexpression of CrtS stimulates the entire carotenogenesis pathway

**Figure 3:**
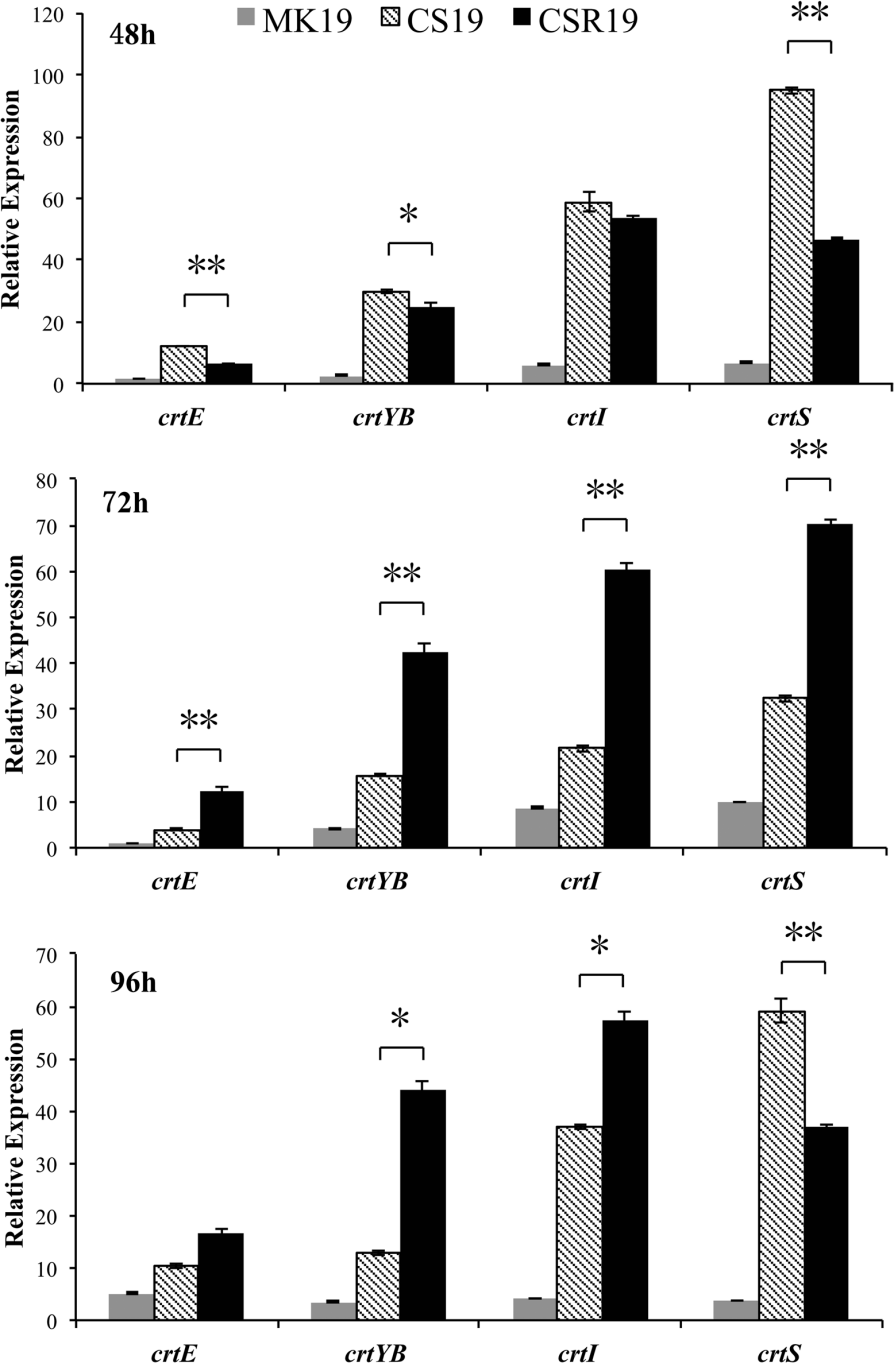
Relative transcription levels of four astaxanthin synthesis-related genes in MK19, CS19, and CSR19 as a function of time. Each strain was flask-cultured for 5 days. *β*-*actin* was used as an internal reference gene for normalization. Expression of *crtE* in MK19 at 48 h was defined as 1, and expression levels of other genes were measured relative to this value. Differences of gene expression between CS19 and CSR19 were analyzed by *t* test, and significant differences are indicated by **(p < 0.01) or *(0.01 < p < 0.05). The *crtS* expression was much higher in CSR19 and CS19 than in MK19. Values shown are means of three individual cultures. *Error bars* SD from three independent biological replicate experiments.

To clarify the regulatory mechanism of carotenogenesis in Crts-overexpressing strains, transcription levels of four genes were studied in CSR19, CS19, and MK19. The genes, all involved in the carotenoid biosynthesis pathway in *P. rhodozyma*, were *crtE* (encoding geranylgeranyl pyrophosphate (GGPP) synthase)*, crtYB* (encoding phytoene synthase)*, crtI* (encoding phytoene desaturase), and *crtS*. Transcription levels at various incubation times are summarized in Figure 3. Expression of *crtS*, the target of overexpression, was strongly enhanced in CSR19 and CS19. Relative to MK19, *crtS* expression was increased consistently for CSR19 (7-fold at 48 h and 72 h, 10-fold at 96 h) and for CS19 (14-fold at 48 h, 3-fold at 72 h, 16-fold at 96 h) throughout the fermentation process. Thus, our overexpression strategy was successful.

Besides the overexpression of *crtS*, mRNA transcription of *crtE, crtYB,* and *crtI* was also much higher in CSR19 and CS19 than in MK19. The first step of the carotenoid pathway is GGPP synthesis catalyzed by CrtE. Overexpression of *crtE* (3- to 12-fold higher in CSR19 and 2- to 9-fold higher in CS19 than in MK19) contributed to increased carotenogenic flux. In many microorganisms, phytoene and lycopene formation are rate-limiting steps in carotenogenesis. Relative to MK19, transcription levels were 9- to 13-fold higher for *crtYB* and 7- to 14-fold higher for *crtI* in CSR19. Similarly, relative transcription levels were 4–11 times higher for *crtYB* and 2–10 times higher for *crtI* in CS19 at various growth stages. These findings indicated that the genes for carotenogenic enzymes are co-regulated, and that genetic manipulation of just one of the genes can induce the entire pathway through step-by-step relief of product feedback inhibition.

The large observed increase in astaxanthin suggested that it was not the inhibitor of this multi-enzyme reaction sequence. Our kinetic studies of mRNA expression patterns of structural carotenogenic genes and their relationship with carotenoid biosynthesis showed significant differences (p < 0.01) between CSR19 and CS19 in expression of the first and last genes responsible for astaxanthin synthesis (*crtE* and *crtS*) during the cell growth period (48 h). When entering the stationary phase (72 h), transcription levels in CSR19 compared with CS19 were 3 times higher for *crtE*, *crtYB*, and *crtI*, and 2 times higher for *crtS* (p < 0.01 in each case). 72 h corresponded to the period of most rapid astaxanthin synthesis (greatest accumulation in CSR19), suggesting that accumulation of sufficient carotenogenic enzymes was necessary for maximal pigment synthesis at the appropriate time. At 96 h, reduction of cell growth and astaxanthin synthesis caused a ~37% down-regulation of *crtS* expression in CSR19 relative to CS19, in contrast to the other three carotenogenic genes which remained more highly expressed in CSR19. In the two transformants, differential expression patterns of the four genes were correlated with differential carotenoid formation patterns.

The ADH1 promoter on pGBKT7 is a modified version of the ADH1 promoter from the yeast *S. cerevisiae*. Its activity increases throughout the yeast growth cycle in both the glucose and ethanol consumption phases. When 300 bp of the upstream sequence was deleted, the promoter became active only in the ethanol consumption phase [18], corresponding to the phase of rapid carotenoid accumulation. Promotion of carotenogenic gene expression at this phase may lead to stimulation of pigment synthesis.

### Structure identification of the unknown carotenoid as 3-hydroxy-3′,4′-didehydro-β,ψ-carotene-4-one (HDCO)

It was not possible to identify the unknown carotenoid unequivocally on the basis of chromatographic properties and absorption spectra data. Therefore high resolution MS (Additional file 4: Figure S3) and NMR (Additional files 5, 6: Figure S4, Table S2) analysis were performed for structural confirmation. High resolution fast-atom bombardment MS gave a quasi-molecular ion peak at *m*/*z* 565.40387 for [M + H]^+^, compatible with the carotenoid formula C_40_H_52_O_2_. Application of MS/MS analysis allowed us to identify ion fragment peaks at* m*/*z* 547.39337 ([M + H–H_2_O]^+^) (Additional file 4: Figure S3). The ^1^H and ^13^C NMR spectral data of the unknown carotenoid were assigned by 2D COSY experiment, which showed it was identical with those of HDCO (Additional files 5, 6: Figure S4, Table S2). It was consistent with the report that HDCO is the major product of the monocyclic carotenoid biosynthesis pathway in *P. rhodozyma* [19].

### Conversion of HDCO to astaxanthin through enhanced CrtS activity

In view of the high production and proportion of astaxanthin obtained in CSR19, we performed single batch fermentation of this strain in a 7.5-L bioreactor. Similar results were obtained in repeated batches. As observed in a representative example (Additional file 7: Figure S5), the exponential phase of cell growth began at 16 h and the stationary phase began at 40 h. The accumulated biomass was maximal (dry weight 24.5 g/L) at the end of the exponential phase (Additional file 7: Figure S5a). Astaxanthin accumulated quickly from 40 to 48 h, and the level did not change notably thereafter. Maximal values were 27.8 mg/L for astaxanthin yield (47.1% higher than for MK19 in the conical flask) and 1,200 μg/g for astaxanthin concentration (Additional file 7: Figure S5b). Dissolved oxygen level was greater in the bioreactor than in the conical flask, enhancing the oxygenase activity of CrtS and the accumulation of astaxanthin and HDCO as oxidation products.

**Figure 4:**
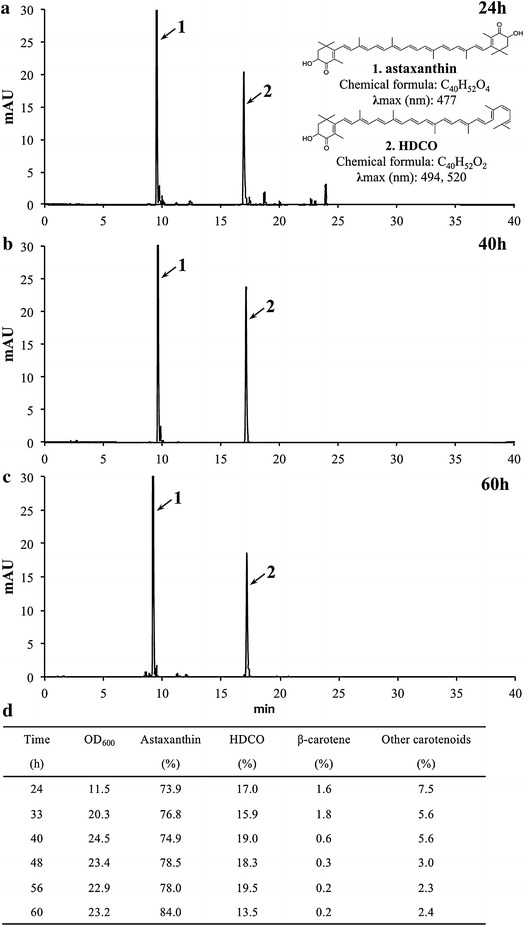
Carotenoid composition of CSR19 during fermentation in 7.5-L bioreactor. HPLC profiles sampling at 24 h (**a**), 40 h (**b**), and 60 h (**c**). Major carotenoid composition monitored at intervals of 8–12 h throughout the culture period (**d**). Detection wavelength: 480 nm. Details of the two major peaks (*1* astaxanthin, *2* HDCO) are shown at *upper right corner*. The maximal astaxanthin peak was normalized as 100 mAU for evaluation of changes in HDCO proportion. The HDCO proportion increased during cell growth (log and exponential phases and declined ~30% in the stationary phase, during which astaxanthin proportion increased accordingly. At 60 h, astaxanthin (84.0%) and HDCO (13.4%) were the only notable carotenoids; others were present in trace amounts.

Carotenoid composition of cultured CSR19 during bioreactor fermentation was evaluated in detail. HPLC profiles of pigments at 24, 40, and 60 h are shown in Figure 4a, b, c. The 24-h sample, besides major peaks 1 (astaxanthin) and 2 (HDCO), has minor peaks corresponding to intermediate products such as keto carotenoids and β-carotene. In contrast, only peaks 1 and 2 are detectable in the 40-h and 60-h samples. The carotenoid profile changed and sampling time details were shown in Figure 4d. The proportion of HDCO relative to total carotenoids increased from 17 to 19% until the stationary phase began at 40 h; thereafter the HDCO proportion declined by 6% while the astaxanthin proportion increased by 6%. Little change in total carotenoid and β-carotene content was observed during the entire conversion process. It is therefore reasonable to assume a relationship between the synthesis levels of HDCO and astaxanthin; the reduced proportion of the former was associated with the increased proportion of the latter (and high CrtS level) during the period of rapid astaxanthin accumulation (40–60 h). At the end of culture, the proportion of astaxanthin was 84% (18% higher than in MK19) and that of HDCO was 13.4%. The only other components were trace carotenoids.

**Figure 5:**
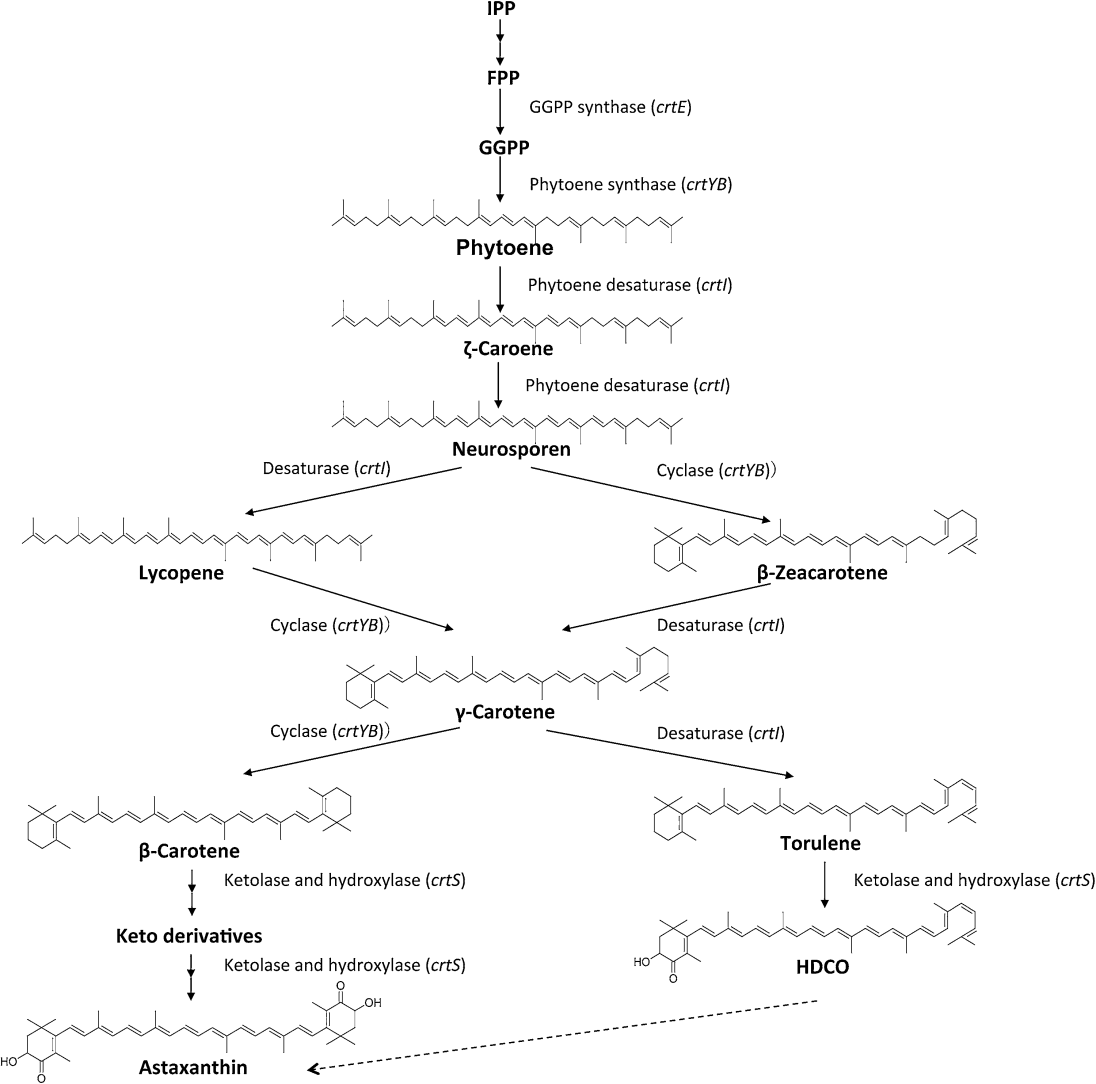
Proposed bicyclic and monocyclic carotenoid biosynthesis pathways in *P. rhodozyma*. The starting compound, acetyl-CoA, passes through the mevalonate pathway for synthesis of isopentenyl pyrophosphate (IPP) and farnesyl pyrophosphate (FPP) as isoprenoid precursors. The following bicyclic pathway involves sequential synthesis of, GGPP, phytoene, lycopene, and β-carotene by a series of catalytic enzymes encoded respectively by *crtE*, *crtYB*, and *crtI*. The recently proposed monocyclic pathway diverges from the bicyclic pathway at neurosporene, with HDCO as the primary product, produced through β-zeacarotene, γ-carotene, and torulene. Results of the present study suggested that CrtS functions in both the bicyclic and monocyclic pathways. Increased levels or activity of CrtS allow HDCO to be used as a substrate for further enhancement of astaxanthin yield (*dashed line* at *bottom*).

A bicyclic carotenogenic pathway in *P. rhodozyma* has been well documented [20–22]. A proposed scheme for astaxanthin synthesis in *P. rhodozyma* based on the present and previous studies is presented in Figure 5. Synthesis starts from acetyl coenzyme A, then enters a terpenoid synthesis pathway [23]. Conversion of isoprenoid precursors into β-carotene occurs through four sequential enzymatic steps, catalyzed by GGPP synthase (encoded by *crtE*), phytoene synthase (encoded by *crtYB*), phytoene desaturase (encoded by *crtI*), and lycopene cyclase (also encoded by *crtYB*) [21, 22]. A monocyclic carotenoid biosynthesis pathway (also shown in Figure 5) was proposed [24]. It diverges from the bicyclic pathway at neurosporene, with HDCO as the primary product, produced through β-zeacarotene, γ-carotene, and torulene.

The *crtS* gene in *P. rhodozyma* was originally reported as encoding a bifunctional hydroxylase/ketolase enzyme responsible for conversion of β-carotene to astaxanthin [14, 15]. Subsequent complementation experiments on *P. rhodozyma* mutants, and expression analysis in *Mucor circinelloides* and *S. cerevisiae*, showed that CrtS has only hydroxylase activity [25]. Based on the results of the present study, we propose that CrtS has bifunctional enzymatic activities in both the bicyclic and monocyclic carotenoid biosynthesis pathways. As indicated by the dashed line at the bottom of Figure 5, carotenoid substrates may be transformed to HDCO as well as astaxanthin during the pigment-accumulating period. HDCO may be converted to astaxanthin at later stages of cell growth, through enhanced CrtS activity.

No standard enzymatic method for detection of CrtS activity has yet been established. The next step in our studies is heterologous expression of the enzyme, to be used as a basis for practical determination of enzyme activity, and further elucidation of enzyme function.

## Conclusions

CrtS appears to be a bifunctional enzyme, responsible for both regulation of carotenoid biosynthesis pathways and conversion of HDCO to astaxanthin. CrtS overexpression leads to up-regulation of synthesis-related genes and increased astaxanthin production. The transformant CSR19 is a stable, secure strain suitable for feed additive production. The present findings help clarify the regulatory mechanisms that underlie metabolic fluxes in *P. rhodozyma* carotenoid biosynthesis pathways.

## Methods

### Strains, plasmids, and culture conditions

Mutant *P. rhodozyma* strain MK19 was generated from wild-type JCM9042 by NTG and Co60 mutagenesis in our previous study [11]. The microbial strains and plasmids used in the present study are listed in Table 2. All strains were maintained on potato dextrose agar (PDA) slants at 4°C.

**Table 2:** Microbial strains and plasmids used in this study

	Description	Source or reference
Strains		
JCM9042	Wild-type strain of *P. rhodozyma*	Institute of Physical and Chemical Research (RIKEN), Japan
MK19	Generated by NTG and Co60 mutagenesis from JCM9042	Miao et al. [11]
CSR19	CrtS-overexpressing strain (with Rbs of *P. rhodozyma crtS* gene)	Present study
CS19	CrtS-overexpressing strain (with Rbs of plasmid pGBKT7)	Present study
*E. coli* DH5α	*EndA1 hsdR17 [r-m*+*] supE44 thi*-*1 recA1 gyrA [NalR]relA1△[lacZYA*-*argF]U169deoR [Φ80△]M15]*	Green et al. [26]
Plasmids		
pGBKT7	Expression vector, ADH1 promoter, Km^r^	Donated by Professor Huiqiang Lou
pGBKT7-*crtSr*	pGBKT7 derivative containing *crtSr* gene (with Rbs of *P. rhodozyma crtS* gene)	Present study
pGBKT7-*crtS*	pGBKT7 derivative containing *crtS* gene (with Rbs of plasmid pGBKT7)	Present study
pMDTM18-T	Cloning vector for gene sequencing, Amp^r^	Takara Co., Japan

Media, seed culture, and flask fermentation were as described previously [11]. Batch fermentation was performed in a 7.5-L bioreactor (Baoxing; Shanghai, China) containing 5 L medium, with initial glucose concentration 60% (w/v), temperature 25°C, pH 5.0, agitation at 700 rpm, and aeration rate 10 L/min. Optical cell density (OD_600_), cell dry weight, astaxanthin production, and glucose consumption were monitored at intervals of 8–12 h throughout the culture period.

### Construction of crtS-overexpressing strains

#### Two kinds of plasmids for expression of crtS

Total RNAs of strains JCM9042 and MK19 were isolated and reverse-transcribed to cDNAs as described previously [27]. The cDNAs were used as templates for cloning of *crtS*. Based on the published *crtS* sequence of *P. rhodozyma* ATCC 24230 (GenBank accession # DQ002006), three primers (termed crtSrCPF, crtSrCPR, crtSCPF) were designed to make two primer sets for specific amplification of MK19 *crtS* sequences with and without ribosome binding site (Rbs). All PCR primer sequences used in this study are listed in Additional file 2: Table S1. Fragments of the expected size (~1.8 kb) were subcloned into vector pMDTM18-T (Takara Co.; Otsu, Japan) and sequenced. Confirmed CrtS cDNA inserts were excised with restriction enzymes *Nco*I and *Pst*I and ligated into *Nco*I and *Pst*I sites of vector pGBKT7 to yield pGBKT7-*crtSr* and pGBKT7-*crtS*, respectively (Additional file 1: Figure S1).

#### Screening and detection of positive transformants

We used a transformation protocol developed previously [20, 28] with several modifications: (1) culture temperature 25°C; (2) harvested cell density (OD_600_) ~ 1; (3) plasmid DNA 4 μL (5 μg/μL) was mixed in 80 μL cell suspension; (4) electroporation (MicroPulser, Bio-Rad; Hercules, CA, USA) parameters: voltage 2,000 V, pulse length 4 ms, electrode gap 0.2 cm.

Plasmids of each transformant were extracted using a yeast plasmid extraction kit (Tiangen Biotech; Beijing, China) and identified by PCR using primers crtSIDF (one of the sequencing primers on pGBKT7) and crtSIDR (whose sequence corresponds to the *crtS* gene). Successful amplification of the expected-length fragment confirmed the *crtS*-overexpressing plasmid. Concentrated plasmids were sequenced for final identification of *crtS* gene expression sequence.

#### Real-time quantitative PCR (qPCR) detection of plasmid copy numbers in transformants

Target plasmid copy numbers in *crtS*-overexpressing strains were determined by qPCR essentially as described by Lee et al. [29]. Two primer sets termed BDQPF-BDQPR and actinQPF2-actinQPR2, specific for plasmid pGBKT7 GAL4 DNA binding domain (DNA-BD) and for *P. rhodozyma* chromosomal *β*-*actin* gene, were used for qPCR analysis. To construct standard sample pGBKT7-*actin*, the *β*-*actin* gene was cloned using actinCPF-actinCPR primer pair, and inserted into plasmid pGBKT7 by restriction enzyme reaction and ligation reaction. Plasmids and genomic DNA (total DNA) of tested strains were prepared using a total DNA extraction method as described previously [26]; chapter on fast separation of yeast DNA. After normalizing, the extracted template DNA was analyzed for quantification in triplicate by qPCR of DNA-BD and *β*-*actin*.

### Transcriptional levels of *crtS* and related genes in astaxanthin synthesis pathway

To detect transcriptional levels of *crtS* and related genes during various cell growth periods, aliquots of cultures of MK19 and its transformants were collected at 24 h (lag phase), 48 h (middle of exponential phase), 72 h (transition from exponential phase to stationary phase), and 96 h (stationary phase) and frozen in liquid nitrogen for subsequent processing. Total RNA isolation and reverse transcription were performed as described in our previous study [25]. qPCR was performed using a LightCycler 480 RT-PCR apparatus and LightCycler 480 SYBR Green I Master Kit (Roche; Mannheim, Germany) according to the manufacturer’s instructions. Genes and primers are listed in Additional file 2: Table S1. *β*-*actin* from *P. rhodozyma* was used as internal control gene. The relative expression of each gene was calculated by the comparative crossing point (Cp) method and presented as 2^−ΔΔCp^. Means were obtained from triplicate analyses.

### Carotenoid extraction and detection

Carotenoids were extracted from MK19 and transformants as described previously [25]. Cells were collected by centrifugation, dispersed by ultrasonication, and disrupted by high-pressure homogenization. Total carotenoids were extracted from cells following step-by-step addition of methanol, acetone/hexane (1:1), and water. The carotenoid-containing upper hydrophobic phase was collected, dried under N_2_ stream, and dissolved in acetone. HPLC analysis of carotenoids was performed on a CBM-20A system equipped with SPD-M20A diode array detector (Shimadzu; Kyoto, Japan). Pigments were separated on a reverse-phase column (C18, 5 μm, 250 × 4.6 mm, Dikma Diamonsil; Lake Forest, CA, USA). Gradient elution system: solvent A = acetonitrile/water 9:1 (v/v); solvent B = ethyl acetate; program 0–25 min, 0–100% B; 25–35 min, 100–0% B; 35–45 min, 0% B isocratic step. Pigment standards were from Sigma.

The unknown carotenoid was isolated by TLC on activated silica plates (Silica Gel 60, 10 × 10 cm, thickness 0.2 mm;, Yantai Chemical Industry Research Institute; Yantai, China), with acetone/hexane (3:7) as mobile phase. Following development, the pink/purple-colored band of the unknown carotenoid was scraped off, eluted with acetone, dried under N_2_ stream, and dissolved in methanol.

A ~100-μg sample was subjected to mass spectrometry on a Thermo Q Exactive high-resolution mass spectrometer (Thermo Scientific; Waltham, MA, USA) in positive mode with conditions: capillary temperature 320°C, spray voltage 3.8 kV, positive ionization, scan range 400–800 *m*/*z*. An NMR spectroscopy sample was prepared by separation through silica gel column chromatography followed by preparative HPLC, and isolated components were identified by NMR spectrometry. ^1^H NMR (500 MHz) and ^13^C NMR (125 MHz) spectra were measured using a UNITY INOVA 500 spectrometer (Varian Inc./Agilent Technologies; Santa Clara, CA, USA) in CDCl_3_ with TMS as an internal standard.

## Additional files

Additional file 1:
**Figure S1.** Structure of vectors used for overexpression of CrtS (astaxanthin synthase) in *P. rhodozyma* strain MK19. a: pGBKT7-*crtSr* containing *P. rhodozyma* *crtS* gene Rbs. b: pGBKT7-*crtS* containing Rbs within pGBKT7 (vector carrying). ADH1 promoter and ADH1 terminator refer to ADH1 from *S. cerevisiae.*
Additional file 2:
**Table S1.** PCR primers used in this study. Additional file 3:
**Figure S2.** Standard curves for *β-actin* (a), GAL4 DNA binding domain (DNA-BD) (b) and Estimated copy numbers (PCN) of two plasmids in strains CS19 and CSR19 (C). A 10-fold serial dilution series of pGBKT7-*actin*, ranging from 1x10^4^ to 1x10^9^ copies/μL, was used to construct standard curves using absolute qPCR in triplicate with BDQP and actinQP2 primer sets. Each curve was generated by plotting Cp values against the logarithm of initial template copy number (n= 2). R^2^: coefficient of determination. Additional file 4:
**Figure S3.** Detection of the unknown carotenoid by high resolution mass spectrometry. High resolution MS analysis showed that the measured mass is 565.40363 for [M+H]^+^, compatible with the chemical formula C_40_H_52_O_2_, and the exact calculated mass is 565.40387. The result of MS/MS showed the fragment ion peaks at m/z 472.33346 ([M-92]^+^), m/z 458.31810 ([M-106]^+^) and m/z 547.39337 ([M+H-H_2_O]^+^). Additional file 5:
**Figure S4.** Detection of the unknown carotenoid by ^1^H NMR (500 MHz) and ^13^C NMR (125 MHz) spectroscopy. The ^1^H and ^13^C NMR spectral data of the unknown carotenoid were assigned by 2D COSY experiment, which showed it was identical with those of HDCO, which is the major product of the monocyclic carotenoid biosynthesis pathway in *P. rhodozyma*. Additional file 6:
**Table S2.** Comparison of ^1^H (500 MHz) and ^13^C (125 MHz) NMR data between the unknown carotenoid and HDCO (2012 report). Additional file 7:
**Figure S5.** Growth (cell dry weight), astaxanthin concentration, and astaxanthin yield as a function of time for CSR19 cultured in a 7.5-L bioreactor. a Cell dry weight was maximal (24.5 g/L) at the end of the exponential phase. b Astaxanthin accumulated quickly from 40 to 48 h, and the level did not change notably thereafter. Maximal values were 27.8 mg/L for astaxanthin yield and 1200 μg/g for astaxanthin concentration.

## Abbreviations

CrtS: astaxanthin synthase
qPCR: real-time quantitative PCR
Rbs: ribosome binding site
GGPP: geranylgeranyl pyrophosphate
HDCO: 3-hydroxy-3′,4′-didehydro-β,ψ-carotene-4-one
