# Supplementary figures and images for: Overexpression of a bifunctional enzyme, CrtS, enhances astaxanthin synthesis through two pathways in Phaffia rhodozyma
Source: Microb Cell Fact. 2015 Jun 18;14:90. doi: 10.1186/s12934-015-0279-4 (PMC4470029; doi:10.1186/s12934-015-0279-4)

**A**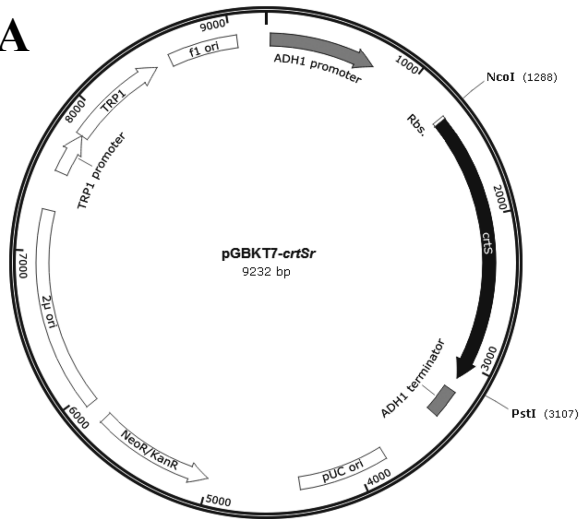**B**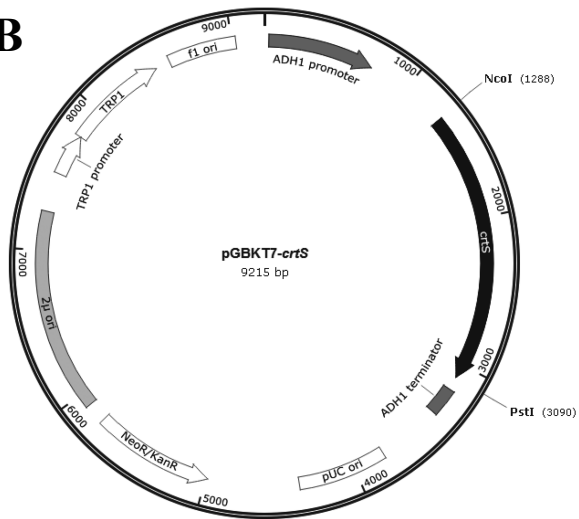

Supplement: Additional file 1: — Figure S1. Structure of vectors used for overexpression of CrtS (astaxanthin synthase) in P. rhodozyma strain MK19. a: pGBKT7-crtSr containing P. rhodozyma crtS gene Rbs. b: pGBKT7-crtS containing Rbs within pGBKT7 (vector carrying). ADH1 promoter and ADH1 terminator refer to ADH1 from S. cerevisiae. [file 12934_2015_279_MOESM1_ESM.pdf]

# MS

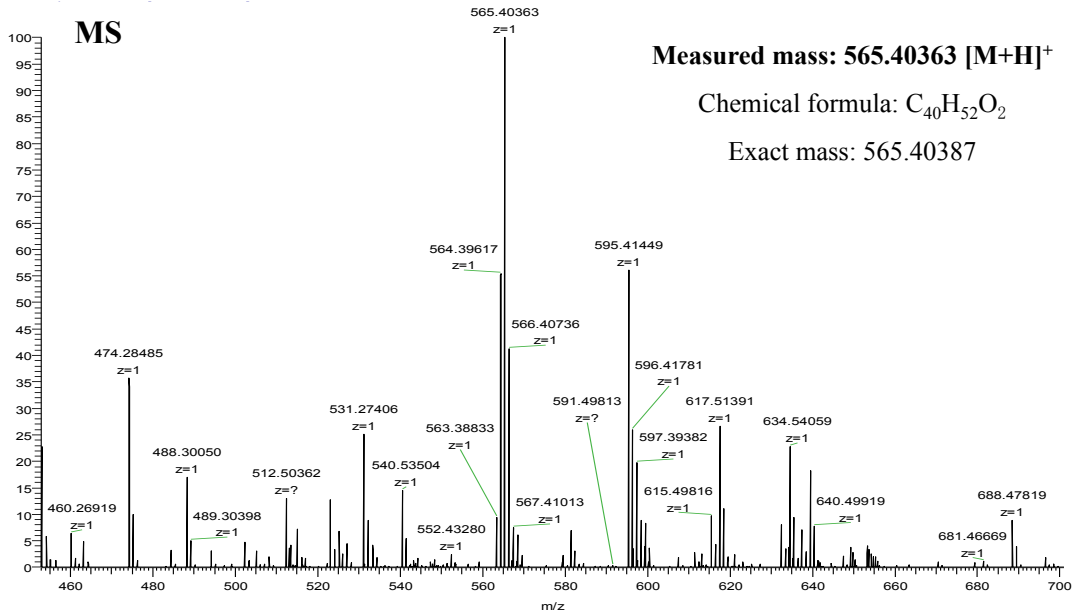

# MS/MS

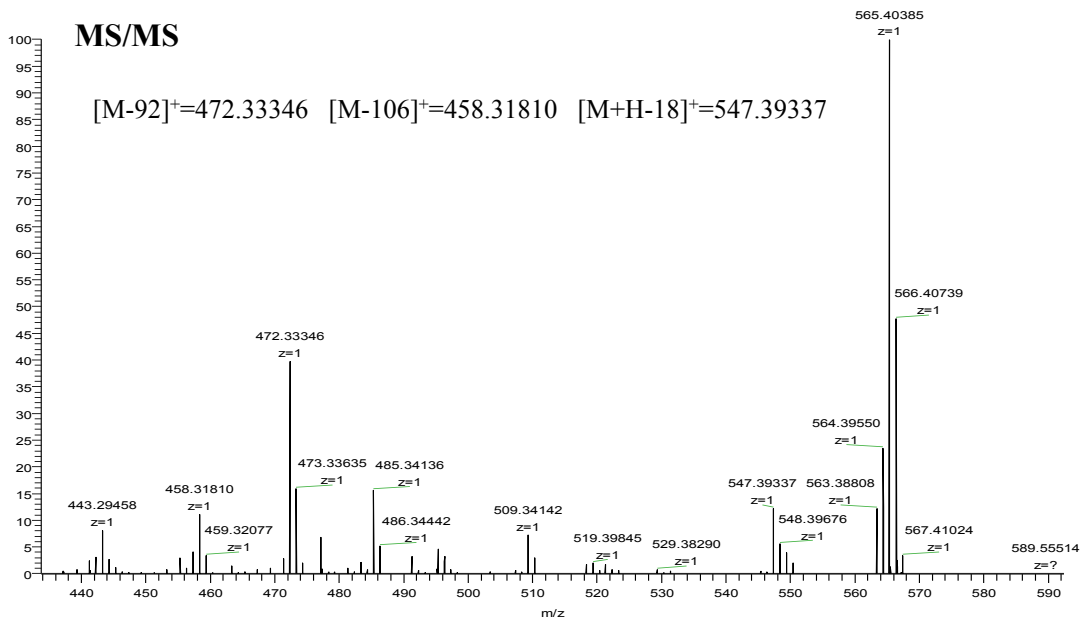

Supplement: Additional file 3: — Figure S2. Standard curves for β-actin (a), GAL4 DNA binding domain (DNA-BD) (b) and Estimated copy numbers (PCN) of two plasmids in strains CS19 and CSR19 (C). A 10-fold serial dilution series of pGBKT7-actin, ranging from 1x10^4^ to 1x10^9^ copies/μL, was used to construct standard curves using absolute qPCR in triplicate with BDQP and actinQP2 primer sets. Each curve was generated by plotting Cp values against the logarithm of initial template copy number (n= 2). R2: coefficient of determination. [file 12934_2015_279_MOESM3_ESM.pdf]

# <sup>1</sup>H (500 MHz) NMR spectrum

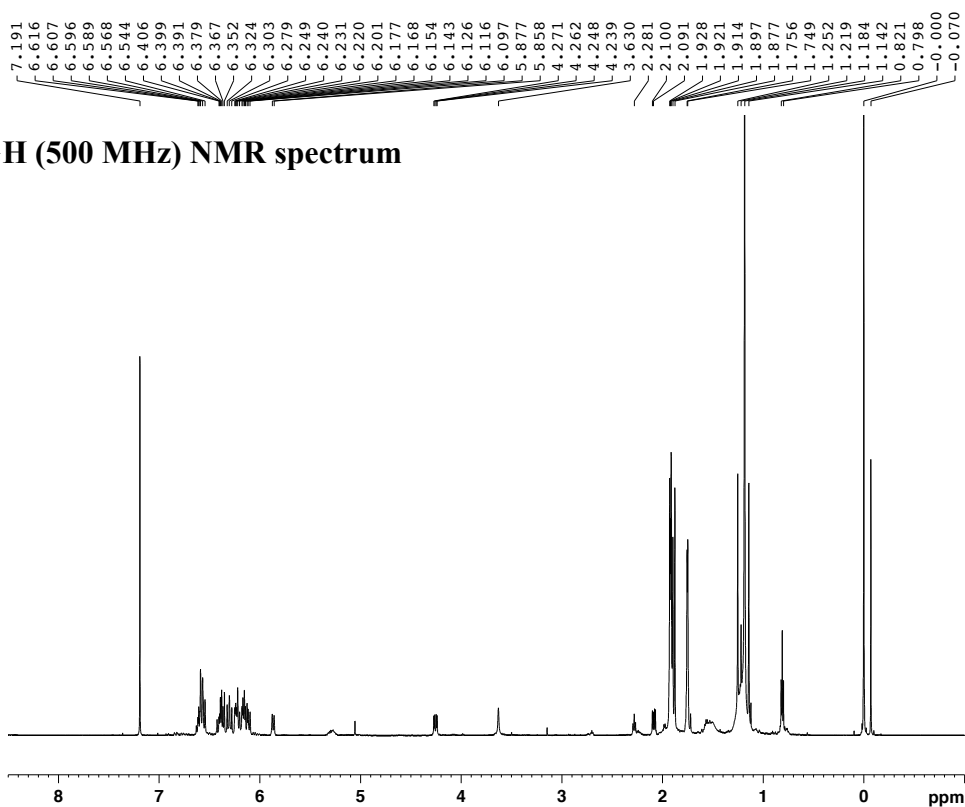

# <sup>13</sup>C (125 MHz) NMR spectrum

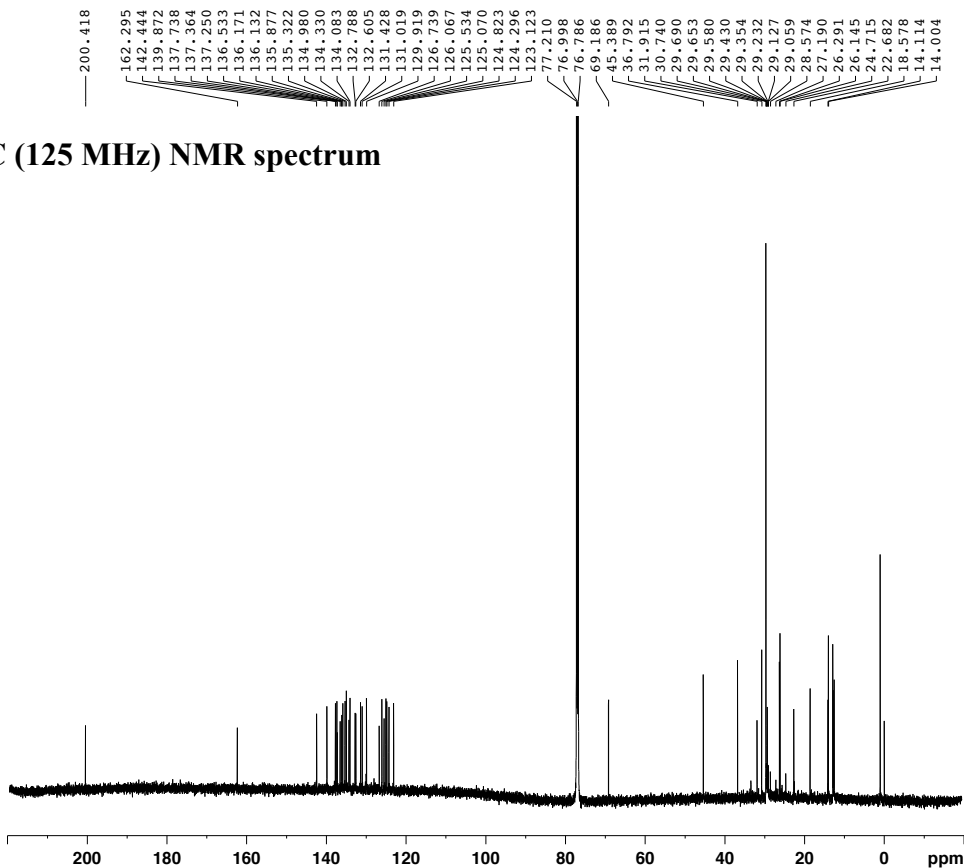

Supplement: Additional file 4: — Figure S3. Detection of the unknown carotenoid by high resolution mass spectrometry. High resolution MS analysis showed that the measured mass is 565.40363 for [M+H]+, compatible with the chemical formula C40H52O2, and the exact calculated mass is 565.40387. The result of MS/MS showed the fragment ion peaks at m/z 472.33346 ([M-92]+), m/z 458.31810 ([M-106]+) and m/z 547.39337 ([M+H-H2O]+). [file 12934_2015_279_MOESM4_ESM.pdf]

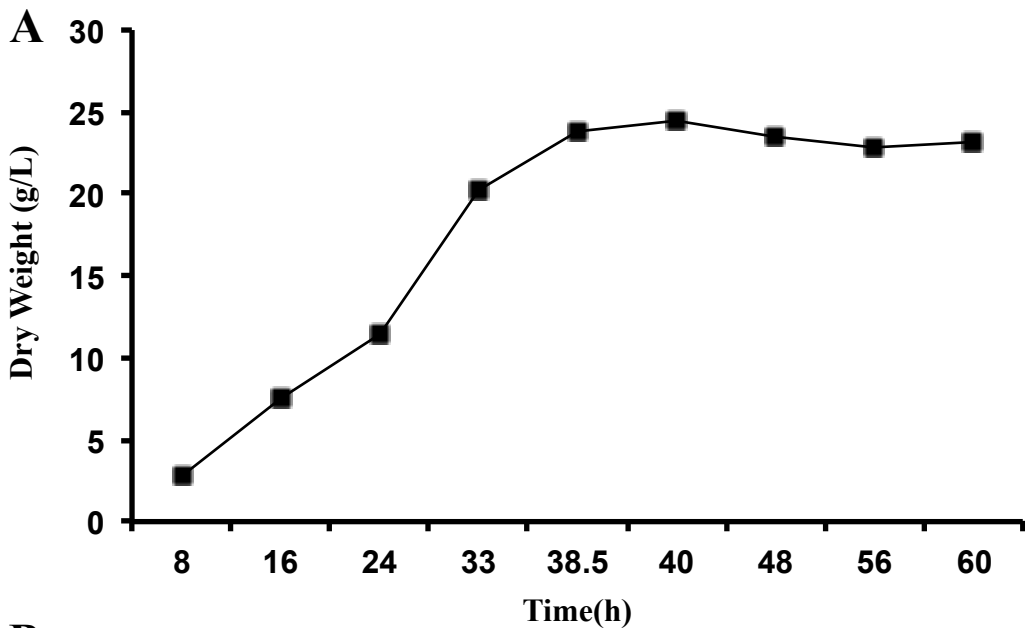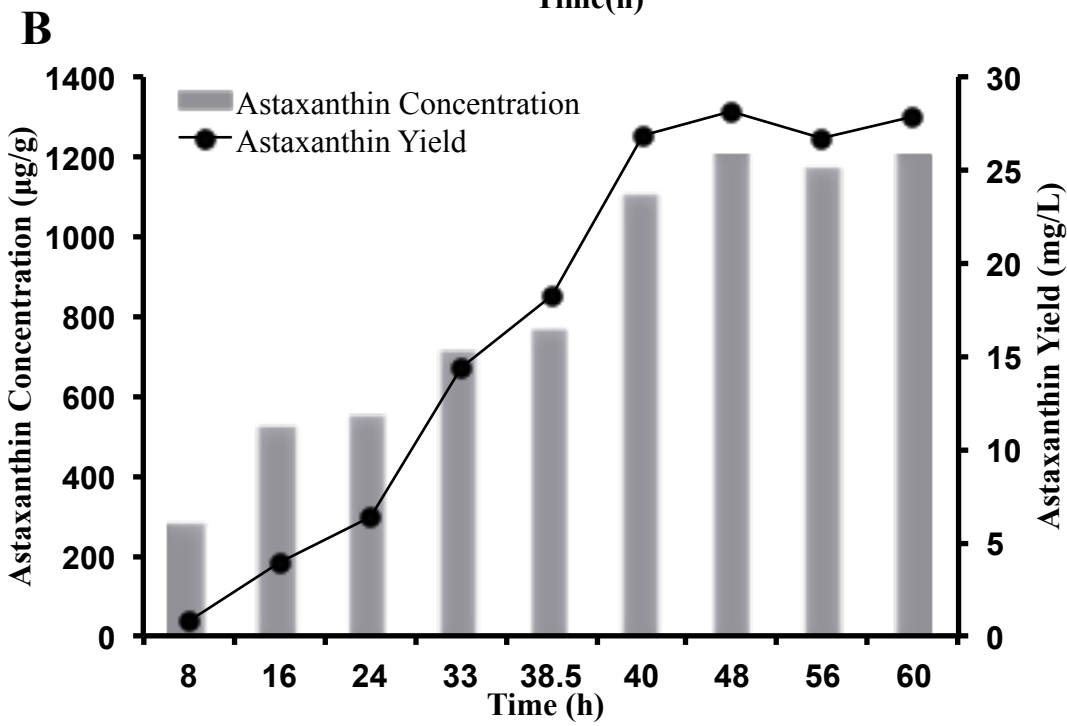

Supplement: Additional file 5: — Figure S4. Detection of the unknown carotenoid by 1H NMR (500 MHz) and 13C NMR (125 MHz) spectroscopy. The 1H and 13C NMR spectral data of the unknown carotenoid were assigned by 2D COSY experiment, which showed it was identical with those of HDCO, which is the major product of the monocyclic carotenoid biosynthesis pathway in P. rhodozyma. [file 12934_2015_279_MOESM5_ESM.pdf]
