# Supplementary material for: Overexpression of a bifunctional enzyme, CrtS, enhances astaxanthin synthesis through two pathways in Phaffia rhodozyma
Source: Microb Cell Fact. 2015 Jun 18;14:90. doi: 10.1186/s12934-015-0279-4 (PMC4470029; doi:10.1186/s12934-015-0279-4)
Supplement: Additional file 2: — Table S1. PCR primers used in this study. [file 12934_2015_279_MOESM2_ESM.pdf]

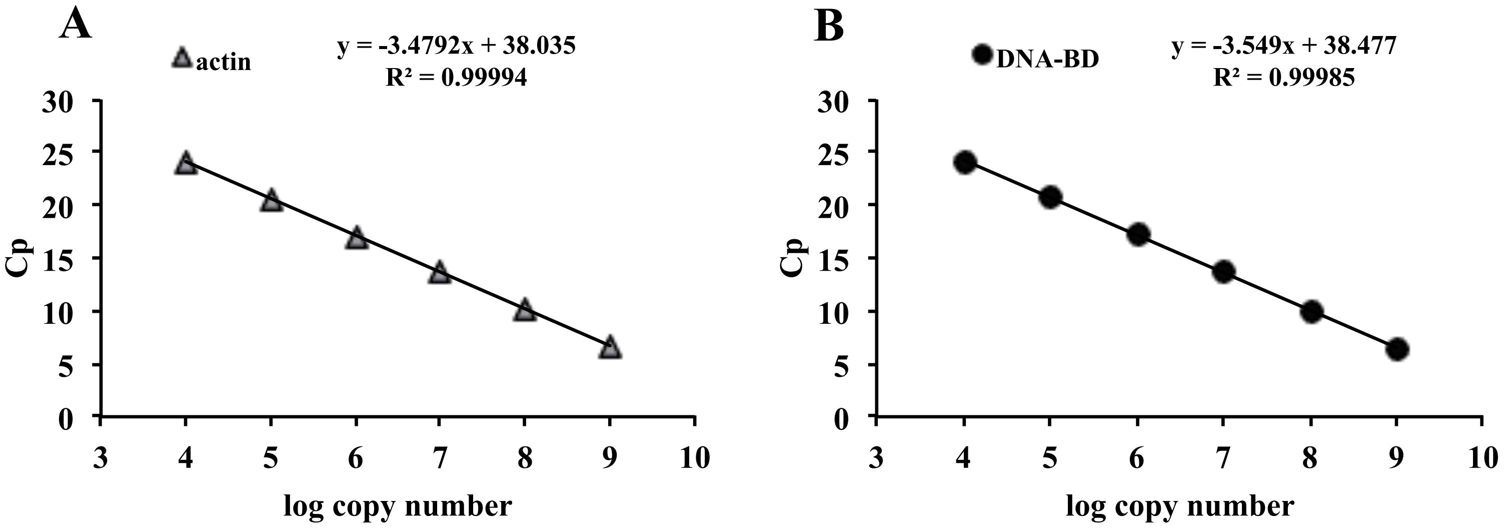

C

Culture	Cp		Copies (copies/µl)		PCN
	DNA-BD	*actin*	DNA-BD	*actin*	
CS19	13.945 ±0.025	17.677 ±0.048	8.166×10^{6}	8.995×10^{5}	9.078
CSR19	14.13 ±0.281	17.707 ±0.012	7.244×10^{6}	8.810×10^{5}	8.222
