# Supplementary material for: Overexpression of a bifunctional enzyme, CrtS, enhances astaxanthin synthesis through two pathways in Phaffia rhodozyma
Source: Microb Cell Fact. 2015 Jun 18;14:90. doi: 10.1186/s12934-015-0279-4 (PMC4470029; doi:10.1186/s12934-015-0279-4)
Supplement: Additional file 6: — Table S2. Comparison of 1H (500 MHz) and 13C (125 MHz) NMR data between the unknown carotenoid and HDCO (2012 report). [file 12934_2015_279_MOESM6_ESM.docx]

**Table S2.** Comparison of ^1^H (500 MHz) and ^13^C (125 MHz) NMR data between the unknown carotenoid and HDCO (2012 report)

| No. | Unknown carotenoid | | | HDCO [Maoka et al., J. Oleo Sci. 61(7): 401-406 (2012)] | | |
| --- | --- | --- | --- | --- | --- | --- |
|  | δ^13^C | δ^1^H | mult.(*J*. Hz) | δ^13^C | δ^1^H | mult.(*J*. Hz) |
| 1 | 36.79 |  |  | 36.8 |  |  |
| 2 | 45.38 | 1.82 | overlapped | 45.4 | 1.82 | overlapped |
| 3 | 69.18 | 2.15 | dd (4.0, 11.0) | 69.2 | 2.15 | dd (15, 3) |
| 4 | 200.41 | 4.32 | dd (4.0, 11.0) | 200.4 | 4.32 | ddd (15, 7, 2) |
| 5 | 126.73 |  |  | 126.9 |  |  |
| 6 | 162.29 |  |  | 162.2 |  |  |
| 7 | 123.12 | 6.16-6.47 | m | 123.1 | 6.22 | d (16) |
| 8 | 142.44 | 6.16-6.47 | m | 142.4 | 6.43 | d (16) |
| 9 | 134.33 |  |  | 134.6 |  |  |
| 10 | 135.32 | 6.16-6.47 | m | 135.3 | 6.30 | d (11.5) |
| 11 | 124.82 | 6.61-6.68 | m | 124.7 | 6.66 | dd (15, 11.5) |
| 12 | 139.87 | 6.16-6.47 | m | 139.9 | 6.45 | d (15) |
| 13 | 136.53 |  |  | 136.7 |  |  |
| 14 | 134.08 | 6.16-6.47 | m | 133.8 | 6.26 | d (12) |
| 15 | 137.25 | 6.61-6.68 | m | 136.9 | 6.63 | m |
| 16 | 30.74 | 1.32 | s | 30.8 | 1.33 | s |
| 17 | 26.29 | 1.21 | s | 26.2 | 1.22 | s |
| 18 | 14.00 | 1.94 | s | 13.9 | 1.94 | s |
| 19 | 12.81 | 1.96 | s | 12.8 | 1.95 | s |
| 20 | 12.89 | 1.98 | s | 12.9 | 1.97 | s |
| 1’ | 135.87 |  |  | 136.0 |  |  |
| 2’ | 131.42 | 5.94 | d (9.0) | 131.3 | 5.94 | d (11.5) |
| 3’ | 125.53 | 6.16-6.47 | m | 125.6 | 6.47 | dd (11.5, 11.5) |
| 4’ | 134.98 | 6.16-6.47 | m | 135.0 | 6.20 | d (11.5) |
| 5’ | 137.36 |  |  | 137.4 |  |  |
| 6’ | 131.42 | 6.16-6.47 | m | 131.3 | 6.18 | d (11.5) |
| 7’ | 126.06 | 6.61-6.68 | m | 126.0 | 6.62 | dd (15, 11.5) |
| 8’ | 137.73 | 6.16-6.47 | m | 137.6 | 6.38 | d (15) |
| 9’ | 136.13 |  |  | 136.3 |  |  |
| 10’ | 129.91 | 6.16-6.47 | m | 130.1 | 6.18 | d (11.5) |
| 11’ | 125.07 | 6.61-6.68 | m | 125.2 | 6.65 | dd (15, 11.5) |
| 12’ | 137.73 | 6.16-6.47 | m | 137.9 | 6.36 | d (11.5) |
| 13’ | 136.17 |  |  | 136.5 |  |  |
| 14’ | 132.78 | 6.16-6.47 | m | 132.7 | 6.26 | d (12) |
| 15’ | 129.91 | 6.61-6.68 | m | 130.1 | 6.63 | m |
| 16’ | 26.29 | 1.82 | s | 26.3 | 1.82 | s |
| 17’ | 18.57 | 1.81 | s | 18.6 | 1.82 | s |
| 18’ | 12.89 | 1.94 | s | 12.9 | 1.94 | s |
| 19’ | 12.78 | 1.99 | s | 12.7 | 1.97 | s |
| 20’ | 12.78 | 1.99 | s | 12.7 | 1.97 | d (2) |
| 3-OH |  | 3.70 | br.s |  | 3.69 | s |

s: singlet; br.s: broad singlet; d: doublet; dd: double doublet; m: multiplet.
