# Supplementary material for: Overexpression of a bifunctional enzyme, CrtS, enhances astaxanthin synthesis through two pathways in Phaffia rhodozyma
Source: Microb Cell Fact. 2015 Jun 18;14:90. doi: 10.1186/s12934-015-0279-4 (PMC4470029; doi:10.1186/s12934-015-0279-4)
Supplement: Additional file 7: — Figure S5. Growth (cell dry weight), astaxanthin concentration, and astaxanthin yield as a function of time for CSR19 cultured in a 7.5-L bioreactor. a Cell dry weight was maximal (24.5 g/L) at the end of the exponential phase. b Astaxanthin accumulated quickly from 40 to 48 h, and the level did not change notably thereafter. Maximal values were 27.8 mg/L for astaxanthin yield and 1200 μg/g for astaxanthin concentration. [file 12934_2015_279_MOESM7_ESM.docx]

| Primer name | Sequence (5’-3’) | Target gene (accession number) | Used for |
| --- | --- | --- | --- |
| crtSrCPF | AATGCCATGGCCACCTACTTTCT CCATATG | *crtS* (DQ002006) | Cloning CrtS cDNAs with Rbs. |
| crtSrCPR | AACTGCAGGACGACGTAGAAGTCATAGC | *crtS* (DQ002006) | Cloning CrtS cDNAs with or without Rbs. |
| crtSCPF | TAGGATCCATATGTTCATCTTGGTCTTGC | *crtS* (DQ002006) | Cloning CrtS cDNAs without Rbs. |
| crtSIDF | GTAATACGACTCACTATAGGGCG | *crtS* (DQ002006) | Transformant plasmid identification |
| crtSIDR | GAGGTAAAGACTGAAGAACGC | *crtS* (DQ002006) | Transformant plasmid identification |
| actinQPF | CTACGTTGGTGACGAGG | *β-actin* (X89898) | qPCR amplification |
| actinQPR | GAGGCGTACAAAGAAAGCAC | *β-actin* (X89898) | qPCR amplification |
| crtEQPF | TTCAGTCTTCTGAGTATGCCC | *crtE* (DQ012943) | qPCR amplification |
| crtEQPR | AACTCTCCTTGAAGCCTCC | *crtE* (DQ012943) | qPCR amplification |
| crtYBQPF | TGTCTGCCTGCGATCATACTC | *crtYB* (AJ133646) | qPCR amplification |
| crtYBQPR | AGATCATCAGTCACCCGGC | *crtYB* (AJ133646) | qPCR amplification |
| crtIQPF | AGGCTTTTGGCAGGTTCCTAAT | *crtI* (Y15007) | qPCR amplification |
| crtIQPR | GCTTCTTTCCACCAACTAAGTC | *crtI* (Y15007) | qPCR amplification |
| crtSQPF | CATCCTCTCAGCTCGTACA | *crtS* (DQ002006) | qPCR amplification |
| crtSQPR | ACCATCGACTTAACGGCC | *crtS* (DQ002006) | qPCR amplification |
| BDQPF | AGTGTCGCTACTCTCCCAAAACCA | DNA-BD | qPCR amplification |
| BDQPR | CAATCTATCT GTGACGGCATCTTTATTC | DNA-BD | qPCR amplification |
| actinQPF2 | AGCACGGAATCGTCACCAA | *β-actin* (X89898) | qPCR amplification |
| actinQPR2 | GTCATCTTCTCTCTGTTAGCCTTG | *β-actin* (X89898) | qPCR amplification |
| actinCPF | AATGCCATGGGTATGGATGACGCAACTG | *β-actin* (X89898) | Cloning *β-actin* cDNA |
| actinCPR | AACTGCAGTGGTGGTGAAAGGGTATC | *β-actin* (X89898) | Cloning *β-actin* cDNA |
|  |  |  |  |

**Table S1.** PCR primers used in this study
